# Exceptional point engineered glass slide for microscopic thermal mapping

**DOI:** 10.1038/s41467-018-04251-3

**Published:** 2018-05-02

**Authors:** Han Zhao, Zhaowei Chen, Ruogang Zhao, Liang Feng

**Affiliations:** 1 Department of Electrical and Systems Engineering, University of Pennsylvania, Philadelphia, PA 19104 USA; 2 Department of Biomedical Engineering, The State University of New York at Buffalo, Buffalo, NY 14260 USA; 3 Department of Materials Science and Engineering, University of Pennsylvania, Philadelphia, PA 19104 USA

## Abstract

Thermal sensing with fine spatial resolution is important to the study of many scientific areas. While modern microscopy systems allow optical detection at high spatial resolution, their intrinsic functions are mainly focused on imaging but limited in detecting other physical parameters, for example, mapping thermal variations. Here, with a coating of an optical exceptional point structure, we demonstrate a low-cost but efficient multifunctional microscope slide, supporting real-time monitoring and mapping of temperature distribution and heat transport in addition to conventional microscopic imaging. The square-root dependency associated with an exceptional point leads to enhanced thermal sensitivity for precise temperature measurement. With a microscale resolution, real-time thermal mapping is conducted, showing dynamic temperature variation in a spatially defined area. Our strategy of integrating low-cost and efficient optical sensing technologies on a conventional glass slide enables simultaneous detection of multiple environmental parameters, producing improved experimental control at the microscale in various scientific disciplines.

## Abstract (web summary)

Thermal imaging of a sample simultaneously with standard microscopy often requires complex modifications to the microscope. Here, Zhao et al. design a simple non-Hermitian structure that can be coated onto a glass slide where exceptional-point enhanced thermal sensing enables this capability.

## Introduction

An optical microscope produces magnified images of different types of specimens, enabling direct observation of microscale topographic details of the objects. It has become a fundamental tool advancing the modern photonic and biological sciences. Beyond conventional optical imaging, real-time inspection and mapping of various physical parameters are also highly desired with microscale spatial resolution^1–4^, such as temperature, humidity, pH, particle impurity and atmospheric pressure. Despite improved spatial resolution for topography-type imaging, it is difficult to directly modify the state-of-the-art microscope systems to accommodate functions of simultaneously mapping other important physical parameters^5–9^. Among many environmental parameters crucial to chemical and biological measurements is temperature^4^. It remains a grand challenge to measure the temperature distribution of a sample with both high sensitivity and high spatial resolution. For example, the spatial resolution of the conventional thermocouples is typically on the order of millimeters^10^, which is too coarse for microscopic temperature detection. The reliabilities of terahertz or infrared thermal mapping techniques are, unfortunately, significantly affected by the reflection from the specimen’s surfaces as it interferes with the optical signal radiated from the heat source^11–14^. Although plasmonic resonant structures have been attempted to characterize thermal perturbations, their raw thermal sensitivity, while enhanced by plasmonic resonance-induced strong field confinement, is still limited by the linear proportion to the perturbation strength^15–18^. Additionally, the required wavelength scanning is time-consuming, preventing further practical applications for efficient real-time monitoring and mapping of the ambient thermal distribution of the target specimen.

Recently, the emergence of non-Hermitian optics has demonstrated a strategic control of light transport by exploring the complex dielectric permittivity in its entirety^19–23^. Non-Hermitian singularities, also known as exceptional points (EPs), can arise, featured by simultaneous coalescence of both eigenvalues and eigenstates in the complex eigen-spectrum. EPs are different from the energy-degenerate diabolic points (DPs) in conventional optical resonant structures that possess only the degenerate eigenvalues. Subject to a weak parametric perturbation, the EP degeneracy can be lifted, resulting in unique eigenvalue splitting proportional to the square root of the perturbation strength. When exploited as a weak transduction signal in sensor applications, such square-root energy splitting represents pronounced enhancement of raw sensitivity superior to the linear response from the DP degeneracy^24–27^.

Here, without modifying the microscope system, we demonstrate a low-cost thermo-sensitive microscope slide for highly distributed temperature mapping and real-time monitoring of heat transport. By utilizing the enhanced sensitivity intrinsically associated with the topology of non-Hermitian EPs, a multilayer EP structure is designed and coated on a conventional microscope slide to sense temperature variations. Under the ambient thermal perturbation, the polymer layer in the EP structure deforms due to the transferred heat, causing the lifting of EP degeneracy and consequently the increase of reflection following a square-root relation. Through the reflection measurements at the initial EP wavelength, the temperature distribution on the glass slide can be gauged with high spatial resolution, facilitating efficient thermography in addition to conventional topographic imaging in an intact microscope system.

## Results

### Design of a thermo-sensitive microscope slide

**Fig. 1:**
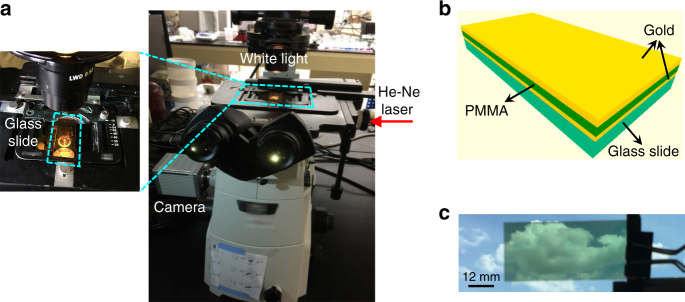
Thermo-sensitive microscope slide engineered at an exceptional point. **a** A microscope system with the devised thermo-sensitive glass slide. While the white light source is for conventional microscopic imaging, the He–Ne laser (labeled by red arrow) is used as the incidence for thermal mapping. Inset: zoom-in of the glass slide in the microscope system. **b** Schematic drawing of the thermo-sensitive glass slide engineered at an exceptional point. A three-layer structure of Au–PMMA–Au is deposited on a silica glass slide. **c** Transparency of the thermo-sensitive glass slide. Revealed by the observable landscape behind the glass slide, the transmission of white light is high enough for optical microscope topographic imaging, while thermal sensing is added as an additional function to the glass slide

The most important feature of a multifunctional slide is its compatibility and easy implementation with a microscope. As shown in Fig. 1a, a thermo-sensitive glass slide is conveniently mounted on a conventional epi-fluorescent microscope system. While maintaining the transmitted-light imaging mode intact, a monochromatic laser light at the EP wavelength is applied to map the reflection variation. As the most cost-efficient and widely used laser source in many routine laboratory settings, a He–Ne laser at the wavelength of 632.8 nm, which can be seamlessly integrated with most microscopes, is chosen in our work to probe the optical-thermal signal transduction.

Upon normal incidence of the He–Ne probe laser on a two-port optical system (note that a microscope slide is a typical two-port system), its optical characteristics can be described using the scattering matrix

1 $$S = \left( {\begin{array}{*{20}{c}} t & {r_{\mathrm{b}}} \\ {r_{\mathrm{f}}} & t \end{array}} \right),$$

where *r*_f_ and *r*_b_ are the reflection coefficients in forward and backward directions, respectively, and *t* denotes the transmission coefficient that is the same in both directions due to reciprocity. The scattering matrix formalism has been previously employed in a multilayer structure to fine-tune and optimize a surface plasmon resonance based sensor^18^. In our work, using the standard optical transfer matrix method for multilayer structures^18,28^ (see Supplementary Note 1), the objective of implementing the scattering matrix is to construct a non-Hermitian EP degeneracy. The eigenvalues of the scattering matrix are $\upsilon _{1,2} = t \pm \sqrt {r_{\mathrm{f}}r_{\mathrm{b}}}$. To achieve the EP where two eigenvalues become degenerate, the eigenvalue splitting has to vanish, i.e., $2\sqrt {r_{\mathrm{f}}r_{\mathrm{b}}} = 0$. Therefore, such coalescence of the scattering eigenstates corresponds to the previously demonstrated unidirectional reflectionless condition: $r_{\mathrm{f}} \ne r_{\mathrm{b}} = 0$^23,29,30^.

To demonstrate a glass slide with such a unidirectional reflectionless EP response, we conduct a 3-layer heterostructure design with a delicate interplay of a thermally deformable polymer layer of polymethyl methacrylate (PMMA) sandwiched between two Au films whose complex refractive index was confirmed at 0.178 + 3.556*i* by ellipsometer measurements (Fig. 1b). By optimizing the thickness of each layer, the EP-supported unidirectional reflectionless light transport is obtained for the probe laser, i.e. at the wavelength of 632.8 nm. At the initial state of room temperature, the EP condition results in complete reflection darkness in the forward direction. Under temperature perturbations, the local thickness of polymer varies due to thermal expansion/suppression, which breaks the EP condition, lifting the EP degeneracy and thus causing a drastically enhanced reflection coefficient. As a consequence, spatially imaging the reflection change is equivalent to resolving and mapping the thermal response of the polymer layer, which can be further converted to a microscopic image of the temperature distribution.

It is important to note that, despite the coating of the multilayer EP structure, the glass slide is transparent under white light illumination, evidently indicated by the visible landscape behind the slide (Fig. 1c). Hence, in addition to the new function of temperature detection, the glass slide maintains the conventional topography-type imaging function of a microscope with transmitted light.

### Sensitivity enhancement by the optical exceptional point

Another critical property of the glass slide is its thermal sensitivity. To precisely map the temperature distribution and variation, it is highly desired to demonstrate high thermal sensitivity. In our designed multilayer EP structure, sensitivity enhancement arises from the intrinsic square-root feature of the scattering eigenvalues of the system when the temperature perturbation drives the system across the EP^26,27^.

**Fig. 2:**
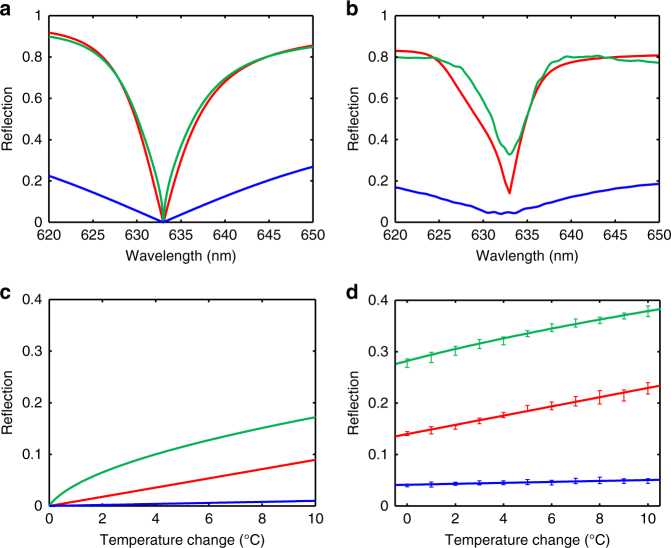
Optical characterization of enhanced thermal sensitivity enabled by exceptional point. **a** Calculated spectra of half the scattering eigenvalue splitting, i.e., the generalized reflection ($\Delta \upsilon {\mathrm{/}}2 = \left| {\sqrt {r_{\mathrm{f}}r_{\mathrm{b}}} } \right|$, green curve) and the forward reflection coefficient (|*r*_f_|, red curve) from the exceptional point (EP) engineered glass slide, which are in stark contrast with the reflection spectrum of the diabolic point (DP) structure with a 1904-nm-thick PMMA anti-reflection film on a glass slide (|*r*|, blue curve). The EP degeneracy featured by zero scattering eigenvalue splitting is designed at 632.8 nm by optimizing the respective thicknesses of Au and PMMA layers. The abrupt EP phase transition can be fully characterized by the quasi-linear response of forward reflection with almost negligible differences. **b** Experimentally measured spectra of the generalized reflection and the forward reflection from the fabricated EP glass slide, and the reflection from the DP structure. The minimum forward reflection at the EP wavelength slightly deviates from the ideal reflectionless condition, and is measured at 0.14 (corresponding to a reflectance of ~2%) due to fabrication imperfection. However, the sharp EP phase transition is still observed. **c** Theoretical temperature responses of scattering eigenvalue splitting and the forward reflection at 632.8 nm, compared with the reflection from the DP structure. Near the EP degeneracy, the scattering eigenvalue splitting evolves with a square-root relation with the temperature increase, providing more than 1 order of magnitude sensitivity enhancement. **d** Calibration of the thermal sensitivity in terms of the generalized reflection as well as the forward reflection. Because of the initial deviation from an ideal EP condition observed in **b**, the slopes of the measured $\left| {\sqrt {r_{\mathrm{f}}r_{\mathrm{b}}} } \right|$ and |*r*_f_| spectra are similar, indicating similar thermal sensitivities for the two cases. Compared with the barely detectable thermal response from the DP control sample, the EP glass slide provides significantly improved thermal sensitivity. Each error bar records the data of five separate measurements and indicates the standard deviation. The curves are the best fits of the medians of these measurements. In the calculation of the thermal response **c**, the empirical thermal expansion of PMMA is taken as *α*_L_=8.2 × 10^−5^ °C^−1^ as a result of experimental data fitted with the theory, where the thermal-induced thickness change is related to temperature variation by Δ*L*=*α*_L_×*L*×Δ*T*

The EP and its associated phase transition are both theoretically and experimentally validated through the amplitude spectrum of the generalized reflection coefficient that is half the scattering eigenvalue splitting: $\Delta \upsilon {\mathrm{/}}2 = \left| {\sqrt {r_{\mathrm{f}}r_{\mathrm{b}}} } \right|$ (Fig. 2a, b). Around the EP, the splitting of the square-root scattering eigenvalue sharpens the phase transition in wavelengths across the EP, which is fundamentally distinguished from the linear variation in its counterpart of Hermitian degeneracies (i.e. DPs, such as zero-reflection obtained from a PMMA anti-reflection film coated on the glass slide) and the trivial metal-induced reflection enhancement (see Supplementary Note 2). Despite a slight discrepancy between the theoretical and experimental results due to fabrication imperfection, the optical response around the EP is much more drastic than that of the DP. Since the implemented multilayer structure is a completely linear system, the direct measurement of the generalized reflection $\left( {\left| {\sqrt {r_{\mathrm{f}}r_{\mathrm{b}}} } \right|} \right)$ and the product of separately measured forward and backward reflections (i.e. $\sqrt {\left| {r_{\mathrm{f}}} \right| \cdot \left| {r_{\mathrm{b}}} \right|}$) are fully equivalent (see Supplementary Note 3 for the experimental details). Such equivalence facilitates the representation of the eigenvalue splitting using only the forward reflection: As a result of the highly asymmetric behaviors of reflections at the EP, the abrupt phase transition across the EP can be clearly revealed if measuring only the forward reflection coefficient |*r*_f_|. This is because the backward reflection is of a large value and varies slowly around the EP, and is therefore not responsible for the sharp transition in the spectrum (see Supplementary Fig. 6 for the calculated and measured spectra of forward and backward reflections, respectively). This observation helps to simplify our experiments significantly. Instead of analyzing the eigenvalue splitting that requires reflection measurements from both directions, only forward reflection needs to be characterized to evaluate all the EP-related properties.

To establish a real-time optical transduction mechanism in thermal sensing, the correlation of the ambient temperature and the scattering eigenvalue splitting is characterized at the EP wavelength. Assume the transferred heat causes the thickness variation of the polymer (PMMA) layer *L* by a sufficiently small deformation Δ*L*. Note that the thickness change of the thin Au layers is negligible. However, the presence of the Au films plays an essential role in creating the unique topology around the EP for the sensitivity enhancement. The scattering matrix subject to the thermal-induced perturbation then becomes

2 $$S = S_0^{{\mathrm{EP}}} + \Delta L\left( {\begin{array}{*{20}{c}} {\frac{{\partial t}}{{\partial L}}} & {\frac{{\partial r_{\mathrm{b}}}}{{\partial L}}} \\ {\frac{{\partial r_{\mathrm{f}}}}{{\partial L}}} & {\frac{{\partial t}}{{\partial L}}} \end{array}} \right) = \left( {\begin{array}{*{20}{c}} {t_0^{{\mathrm{EP}}} + \Delta L\frac{{\partial t}}{{\partial L}}} & {r_{\mathrm{b}}^{{\mathrm{EP}}} + \Delta L\frac{{\partial r_{\mathrm{b}}}}{{\partial L}}} \\ {r_{\mathrm{f}}^{{\mathrm{EP}}} + \Delta L\frac{{\partial r_{\mathrm{f}}}}{{\partial L}}} & {t_0^{{\mathrm{EP}}} + \Delta L\frac{{\partial t}}{{\partial L}}} \end{array}} \right),$$

where $r_{\mathrm{f}}^{{\mathrm{EP}}} = 0$ and $r_{\mathrm{b}}^{{\mathrm{EP}}} \ne 0$, referring to the initial EP unidirectional reflectionless condition. Thermal deformation lifts the EP degeneracy with a scattering eigenvalue splitting of

3 $$\Delta \upsilon = 2\sqrt {\Delta L\frac{{\partial r_{\mathrm{f}}}}{{\partial L}}\left( {r_{\mathrm{b}}^{{\mathrm{EP}}} + \Delta L\frac{{\partial r_{\mathrm{b}}}}{{\partial L}}} \right)} .$$

Because $\left| {r_{\mathrm{b}}^{{\mathrm{EP}}}} \right|$ is of a large value and remains almost a constant at the EP with respect to the temperature increase (see Supplementary Fig. 7), the square-root relation is revealed as $\Delta \upsilon ^{{\mathrm{EP}}} = 2\sqrt {\frac{{\partial r_{\mathrm{f}}}}{{\partial L}}r_{\mathrm{b}}^{{\mathrm{EP}}}} \cdot \sqrt {\Delta L}$, where the sensitivity can be fully characterized by only the derivative of forward reflection. As a result, the thickness dependence of the eigenvalue splitting is mainly on the change of forward reflection, while the relatively large backward reflection at the EP acts as a magnifying factor. Note that for a DP degeneracy, Eq. (3) evolves to a linear relation with the thickness perturbation of $\Delta \upsilon ^{{\mathrm{DP}}} = 2\Delta L\frac{{\partial r_{\mathrm{f}}}}{{\partial L}}$ since the Hermitian condition regulates simultaneous reflection vanishing in both directions (i.e., $r_{\mathrm{b}}^{{\mathrm{DP}}} = r_{\mathrm{f}}^{{\mathrm{DP}}} = 0$). Such a striking contrast of the eigenvalue splitting between the designed EP structure and a typical DP structure (such as the PMMA anti-reflection film studied in our work) reads

4 $$\frac{{\Delta \upsilon ^{{\mathrm{EP}}}}}{{\Delta \upsilon ^{{\mathrm{DP}}}}} = \sqrt {\Delta L} \frac{{\sqrt {\left( {\frac{{\partial r_{\mathrm{f}}}}{{\partial L}}} \right)_{{\mathrm{EP}}}r_{\mathrm{b}}^{{\mathrm{EP}}}} }}{{\left( {\frac{{\partial r_{\mathrm{f}}}}{{\partial L}}} \right)_{{\mathrm{EP}}}}}.$$

Under weak perturbations, the relation of $(\partial r_{\mathrm{f}}/\partial L)_{{\mathrm{EP}}}r_{\mathrm{b}}^{{\mathrm{EP}}} > > \left[ {(\partial r_{\mathrm{f}}/\partial L)_{{\mathrm{DP}}}} \right]^2$ensures significant sensitivity improvement in terms of per unit change of the PMMA thickness. Specifically, starting from the degeneracy points, the unit temperature change of 1 °C leads to the EP splitting of $\Delta \upsilon ^{{\mathrm{EP}}} = 0.0818$ and $2\Delta \left| {r_{\mathrm{f}}} \right| = 0.019$, both offering 1 order of magnitude enhancement compared with the merely detectable DP splitting of $\Delta \upsilon ^{{\mathrm{DP}}} = 0.002$ under the same thermal perturbation, assuming the same thermal expansion of PMMA at 8.2 × 10^−5^ °C^−1^ (Fig. 2c). Figure 2d shows the measured and calibrated relations of the temperature variation with half the scattering eigenvalue splitting, i.e., the generalized reflection $\left| {\sqrt {r_{\mathrm{f}}r_{\mathrm{b}}} } \right|$ as well as only the forward reflection |*r*_f_| from a He–Ne probe laser. We note that the non-zero scattering eigenvalue splitting at room temperature due to fabrication imperfection leads to similar attainable sensitivity compared with measuring only the forward reflection signal. The observed enhanced thermal sensitivity is 0.0172 ± 0.0016 °C^−1^ in a 10 °C range, which is ~1 order of magnitude better than 0.00187 ± 0.00043 °C^−1^ obtained from the DP control sample. To maintain a high sensitivity in a maximum dynamic range, it is desired to reach the ideal degeneracy for |*r*_f_|=0. While this is achievable with both the EP and DP, the good sensitivity is only associated with the EP according to aforementioned discussions, which enables precise readings of temperature on the glass slide. Additionally, the sensitivity enhancement by our multilayer EP structure also holds when compared with a trivial symmetric metal–polymer–metal sandwiched structure where the condition of |*r*_f_|=0 or |*r*_b_|=0 cannot be easily satisfied (i.e., no EP or DP can be achieved) (see Supplementary Note 2).

It is important to note that the dynamic range and the sensitivity are often a pair of trade-off parameters in sensors. While the drastic sensitivity improvement can be observed close to the EP (±2 nm from the EP), our thermo-sensitive microscope slide can meet the needs for most biological applications where the temperature variation is expected to vary from room temperature to 43 °C. This range of temperature corresponds to maximally an increase of 0.18 in forward reflection at the EP wavelength and/or a shift of 1.05 nm of the EP resonance, and thus does not require a large dynamic range.

### Spatially resolved thermal mapping

**Fig. 3:**
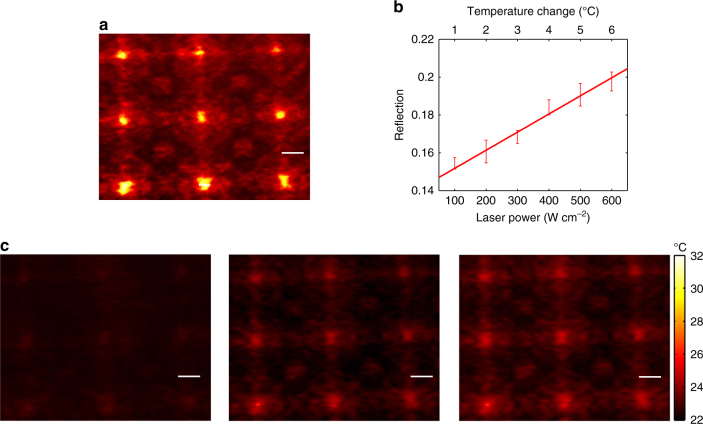
Thermal mapping of spatially resolved pulsed laser heating source. **a** A spatially distributed thermal source array of 3×3 spots revealed by transmission of the incident 1064 nm pulsed laser beam on the glass slide. The laser spots with a diameter averaged at 30 μm are created by imaging a square-latticed hole array on the central PMMA layer through a demagnifying optical setup. As the glass slide absorbs the incident power of the pulsed laser beam, the absorbed heat thermally transfers in the PMMA layer and locally raises the temperature, which results in expansion of PMMA and the lifting of the exceptional point (EP). **b** The correlation of the power density of the pulsed laser beam and the forward reflection of the probe He–Ne laser beam measured at the center spot. With increasing incident power of the heating source, forward reflection of probe He–Ne laser grows linearly from the minimum at the EP condition. Each error bar indicates the standard deviation of three separate measurements, and the line fits the medians of these measurements. **c** Spatially resolved thermal mappings of the heating laser spot array revealed by forward reflection of He–Ne laser beam. Panels from left to right are obtained at increasing power density of the pulsed laser beam at 100 W cm^−2^, 300 W cm^−2^, and 500 W cm^−2^, respectively. Scale bars in **a**, **c** 50 μm

The purpose of the thermal mapping is to retrieve detailed temperature information of the specimen, along with conventional microscopic imaging, to enable the analysis of the correlation between multiple physical parameters. To meet this objective, the desired technique must support thermal mapping, operated in a highly distributed manner with high spatial resolution comparable to the resolution of the microscope. The ultimate spatial resolution of thermal mapping is only limited by the heat transfer-induced spatial temperature overlapping of adjacent heat sources^31^, and is evaluated to be at a 10-μm scale in our device (see Supplementary Note 4). Here, we have validated such a highly distributed thermal mapping function of our microscope slide and its associated microscale spatial resolution. Spatially distributed local thermal sources were effectively generated by optically casting and focusing a 3×3 square-latticed hole array onto the central layer of PMMA to locally heat up and expand the polymer through the backside of the slide. To avoid the interference with the visible measurements, we used a 10-nanosecond pulsed laser with a center wavelength of 1064 nm. Through demagnification (see Supplementary Note 5 for the details of the experimental setup), 9 microscale laser spots were created with a spot diameter averaged at 30 μm, as shown by the corresponding transmission image at the wavelength of 1064 nm (Fig. 3a). While only a small fraction of laser light can be absorbed by the polymer, the heat accumulated from absorption still induces a temperature increase around those 9 spots, which locally enlarges the thickness of the central polymer layer. Due to the heat transfer in the EP multilayer structure, the absorbed heat from laser spots spreads and raises the temperature evanescently within the vicinity of ~4 μm (see heat transfer simulations in Supplementary Fig. 8). As a result, only the local condition around the laser spots deviates from the EP condition designed for room temperature, causing the reflection variation of the probe light from the He–Ne laser by which the temperature change in the vicinity of the laser spots can be mapped. To completely eliminate the adverse effect on thermal mapping due to the transmission of the heating laser beam, a bandpass filter at 500–700 nm was placed in front of the camera to capture only the reflected probe light of the He–Ne laser beam to precisely read the thermal distribution on the microscope slide. It is worth noting that the accumulated heat as well as the resulting variation of the local temperature and the deformation of the polymer layer are all linearly dependent on the power of the incident pulsed laser. Therefore, by varying the power of the pulsed laser, we observed a linear growth in forward reflection of the probe light, which was further converted to a function of temperature through the calibrated temperature–reflection correlation (Fig. 3b). The thermal maps retrieved from the probe light are displayed in Fig. 3c with different averaged incident heating laser power. Remarkably, the imaged thermal distributions spatially resolve the pattern of the heating pulsed laser spot with feature size at 30 μm.

### Real-time mapping of thermal conduction

Another technical advantage of the EP-engineered microscope slide is its high temporal-resolution for real-time monitoring of temperature distribution and evolution in samples. Since many temperature-critical processes occur under a solvent environment^32–34^, we mimicked such an environment by injecting hot water on the glass slide, and experimentally showed the dynamic evolution of the water temperature during heat diffusion into the surroundings.

**Fig. 4:**
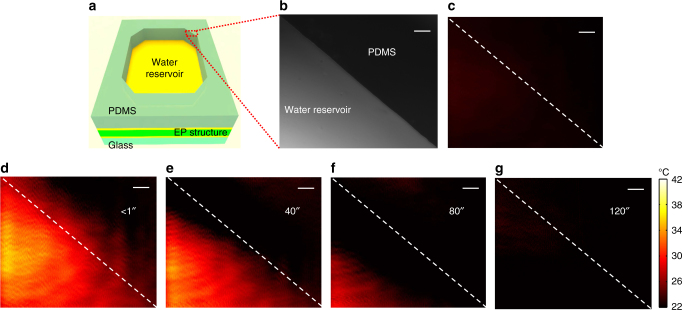
Transient thermal mapping of hot water injection. **a** Schematic drawing of the thermo-sensitive microscope slide with a bonded PDMS octagonal water reservoir for hot water injection. **b** Microscope image of the top right corner of the water reservoir by transmitted white light. The boundary divides the water and PDMS regions, demonstrating transmitted light imaging capacity of the glass slide. **c** Thermal mapping of the reservoir edge area when the reservoir is filled with room temperature water as a control experiment. In the absence of heat transfer, the uniform temperature distribution from detected reflection reveals the initial exceptional point (EP) condition in both water and PDMS regions. **d**–**g** Transient thermal mappings at different time using forward reflection of the He–Ne laser beam, after injection of 43 °C hot water. The instantaneous jump of He–Ne laser reflection in the reservoir and the evanescent tail towards the PDMS region evidently show the in-plane heat transfer process. As the heat diffuses, the detected reflection decreased gradually anti-parallel to the horizontal heat fluxing direction from water to PDMS until the uniform map at thermal equilibrium reached 2 minutes after the water injection, manifesting the evolution of spatial temperature distribution in real time. Dashed lines in **c**–**g** represent the boundary of water and PDMS. Scale bars in **b**–**g**, 100 μm

To confine hot water in a local area for imaging, a water reservoir was built on the slide using a block of polydimethylsiloxane (PDMS) with a hollow octagonal cavity in the middle, which was then bonded on top of the layered EP structure, as schematically depicted in Fig. 4a. Following our aforementioned discussion, while the thermal mapping is performed in a reflection mode mapping the temporal evolution of the temperature distribution of injected water, the slide is still transparent to support microscopic imaging using white light in the transmitted-light mode, where the boundary of PDMS divides the entire field into two regions: an area in the reservoir where hot water injection induces the temperature change and the other area of stabilized temperature covered by PDMS (Fig. 4b). Note that since the top cladding changes from air to water after the hot water injection, the original EP design becomes invalid such that we reconfigure the multilayer structure to support zero-forward reflection for the probe light to function in the EP condition (see Supplementary Fig. 10).

As a control experiment, room temperature water was first injected into the reservoir. Due to their similar refractive indices, water and PDMS provide almost equivalent cladding environments. In this case, the temperature of the glass slide surface remains at room temperature during the water injection. The detected reflection in the field of view stays uniform at its resonance minimum of the designed EP condition in both water and PDMS regions (Fig. 4c).

To observe temporal evolution of the heat transfer process, we injected hot water at ~43 °C and real-time monitored and mapped the temperature distribution. Figure 4d–g show the measured instantaneous thermal maps at different time delays. Right after injecting a droplet of hot water, because of high thermal conductivity of the thin Au layer, the PMMA layer underneath the water reservoir region immediately responds with thermal expansion. As a consequence, the detected reflection significantly increases from the EP resonance minimum, indicating the corresponding local temperature change. Nevertheless, only a negligible amount of heat from hot water can horizontally transfer through the PDMS wall of much lower thermal conductivity, barely varying the thickness of the PMMA layer under PDMS. This leads to an evanescent decay of thermal perturbation across the edge of the reservoir, creating a spatial temperature gradient. As the heat dissipates in the flow direction from the water reservoir to the PDMS cladding until the thermal equilibrium condition is reached, the temperature of water keeps dropping and so does the temperature of the PMMA layer, leading to the contraction of the PMMA layer back to the initial EP condition and its resulting decrease in reflection back to the initial resonance EP minimum. This process has been dynamically recorded (see Supplementary Fig. 10), in which the spatial temperature gradient gradually retracts in time towards the center of the reservoir. Here, the designed thermo-sensitive microscope slide successfully enables real-time monitoring of the dynamic evolution of a heat transfer process, promising in-situ control of temperature in the applications where temperature monitoring is necessary. For example, it may play an important role in protein biosynthesis where enzyme activities are considerably affected by heat generation^35,36^.

## Discussion

In summary, utilizing the enhanced sensitivity arising from an optical EP, we have demonstrated a novel thermo-sensitive glass slide facilitating simultaneous microscopic mapping and monitoring of temperature distribution and thermal variation. The results evidently show that the microscope slide functions as an efficient temperature monitor with enhanced thermal sensitivity and microscale spatial resolution. The described optical transduction mechanism can revolutionize the functionality of ubiquitous glass slides beyond their conventional mechanical supporting of specimen, promising a low-cost microscope-integrated sensing technology for improved controllability of multiple environmental parameters. For example, the glass slide can be further explored to spectrally host two (or multiple) EPs that can respond to temperature (through thermal expansion), pressure (through elastic deformation), and/or other parameters distinctively, formulating a set of independent equations with two (or multiple) variables for simultaneous multi-parameter sensing.

## Methods

### Fabrication

The first layer of Au was deposited on a glass slide using electron beam evaporation. The samples were then spin-coated with PMMA, followed by the second electron beam evaporation of Au. The thicknesses of two Au layers were set to be 23.2 nm and 26.8 nm for the samples in Figs. 1–3 where the medium on top of the EP structure is air, and 21.4 nm and 25.9 nm for the PDMS bonded samples in Fig. 4 where a water cladding is considered to achieve the EP, respectively. The thickness of spin-coated PMMA layer was designed to reach 1840 ± 10 nm controlled with a proper rotation speed. All thicknesses were verified by ellipsometry measurements. The complex refractive index of Au was also calibrated at 0.178 + 3.556*i* using ellipsometry measurements.

### Calibration of temperature–reflection correlation

During the device calibration, the temperature on surface of the glass slide was controlled by a thermoelectric-heating device that was placed 2 cm away from the sample. Due to high thermal conductivity of the Au film, heat was transferred to PMMA, inducing thermal expansion and thus the reflection change due to the lifting of the EP degeneracy. By assuming a thermal equilibrium condition on the multilayer structure, the temperature of the PMMA layer was gauged by a thermocouple with a point contact on the backside of the Au film. The reflection of the He–Ne laser incidence corresponding to the calibrated temperature was then monitored by the charge-coupled device camera.

### Data availability

The data sets within the article and Supplementary Information of the current study are available from the authors upon request.

## Electronic supplementary material

Supplementary Information
