# Supplementary material for: Exceptional point engineered glass slide for microscopic thermal mapping
Source: Nat Commun. 2018 May 2;9:1764. doi: 10.1038/s41467-018-04251-3 (PMC5931976; doi:10.1038/s41467-018-04251-3)
Supplement: Supplementary file 1 — Supplementary Information [file 41467_2018_4251_MOESM1_ESM.pdf]

Supplementary Information

Exceptional Point Engineered Glass Slide for Microscopic Thermal Mapping

Han Zhao[1], Zhaowei Chen[2], Ruogang Zhao[2]* and Liang Feng[3]*

[1]*Department of Electrical and Systems Engineering, University of Pennsylvania, Philadelphia, PA 19104, USA*

[2]*Department of Biomedical Engineering, The State University of New York at Buffalo, Buffalo, NY 14260, USA*

[3]*Department of Materials Science and Engineering, University of Pennsylvania, Philadelphia, PA 19104, USA*

*Emails: fenglia@seas.upenn.edu (L.F.); rgzhao@buffalo.edu (R.Z.)

Supplementary Note 1: Calculation of the scattering matrix by the optical transfer matrix method. We utilize the optical transfer matrix method to determine the scattering matrix of the multi-layer glass slide[1]. The light propagation in each layer of the glass slide can be characterized by

$$M_j = \begin{pmatrix} \cos k_j L_j & \sin(k_j L_j)/k_j \\ -k_j \sin k_j L_j & \cos k_j L_j \end{pmatrix}, \tag{1}$$

where the indices $j = 1, 2, 3$ denote the Au, PMMA, and Au layers, k_j and L_j are the corresponding wavenumber and thickness of each layer (see Supplementary Table 1 for the refractive indices of the materials). The total transfer matrices of the layered structure for light propagation in the forward and backward directions can be derived as

$$M^{\mathrm{f}} = M_3 \cdot M_2 \cdot M_1, \quad M^{\mathrm{b}} = M_1 \cdot M_2 \cdot M_3. \tag{2}$$

The corresponding reflection and transmission coefficients which compose the scattering matrix then read

$$t = 2ik_{\mathrm{l}} e^{-ik_{\mathrm{r}}L} \left[\frac{1}{-M_{21}^{\mathrm{f}} + k_{\mathrm{l}} k_{\mathrm{r}} M_{12}^{\mathrm{f}} + i(k_{\mathrm{r}} M_{11}^{\mathrm{f}} + k_{\mathrm{l}} M_{22}^{\mathrm{f}})} \right], \tag{3}$$

$$r_{\mathrm{f}} = \frac{M_{21}^{\mathrm{f}} + k_{\mathrm{l}} k_{\mathrm{r}} M_{12}^{\mathrm{f}} + i(k_{\mathrm{l}} M_{22}^{\mathrm{f}} - k_{\mathrm{r}} M_{11}^{\mathrm{f}})}{-M_{21}^{\mathrm{f}} + k_{\mathrm{l}} k_{\mathrm{r}} M_{12}^{\mathrm{f}} + i(k_{\mathrm{r}} M_{11}^{\mathrm{f}} + k_{\mathrm{l}} M_{22}^{\mathrm{f}})}, \tag{4}$$

$$r_{\mathrm{b}} = \frac{M_{21}^{\mathrm{b}} + k_{\mathrm{l}} k_{\mathrm{r}} M_{12}^{\mathrm{b}} + i(k_{\mathrm{l}} M_{22}^{\mathrm{b}} - k_{\mathrm{r}} M_{11}^{\mathrm{b}})}{-M_{21}^{\mathrm{b}} + k_{\mathrm{l}} k_{\mathrm{r}} M_{12}^{\mathrm{b}} + i(k_{\mathrm{l}} M_{11}^{\mathrm{b}} + k_{\mathrm{r}} M_{22}^{\mathrm{b}})}, \tag{5}$$

where L is the total length of the three-layer structure, k_{l} is the wave number in thick silica glass as the entering medium for forward light propagation, and k_{r} is the wave number of the air.

Supplementary Note 2: Comparison of sensitivity between EP and metal-induced reflection enhancement. We note the degeneracy ($|r_f| = 0$) of our multilayer scattering system with non-vanishing Au layers is inherently a non-Hermitian exceptional point (EP), while a symmetric Hermitian configuration with metal-enhanced reflectivity cannot reach the diabolic point (DP). To justify this, we note the prerequisite of a DP is a Hermitian scattering system ($|r_f| = |r_b|$) where the polymer layer is sandwiched by two symmetric Au layers of the same thickness. We plot in Supplementary Fig. 1 the calculated reflection of the Hermitian system with variable thicknesses of the polymer layer and the Au layers, which shows the resonance of such a Hermitian system can reach the DP degeneracy ($|r_f| = 0$ and $|r_b| = 0$) only when the Au layers completely vanish. Hence, it is obvious that: 1) if one likes to compare our EP scheme with a DP scheme, one has to reach a control sample of a PMMA anti-reflection film; 2) if one likes to compare our scheme with a Hermitian multilayer structure of finite Au, the Au layers enhance reflectivity such that the reflection resonance can never reach 0. In fact, in a Hermitian system, a larger Au layers leads to increasing reflection at resonance. In contrast, the judiciously designed Au layers in our non-Hermitian multilayer structure vanish the reflection in one direction.

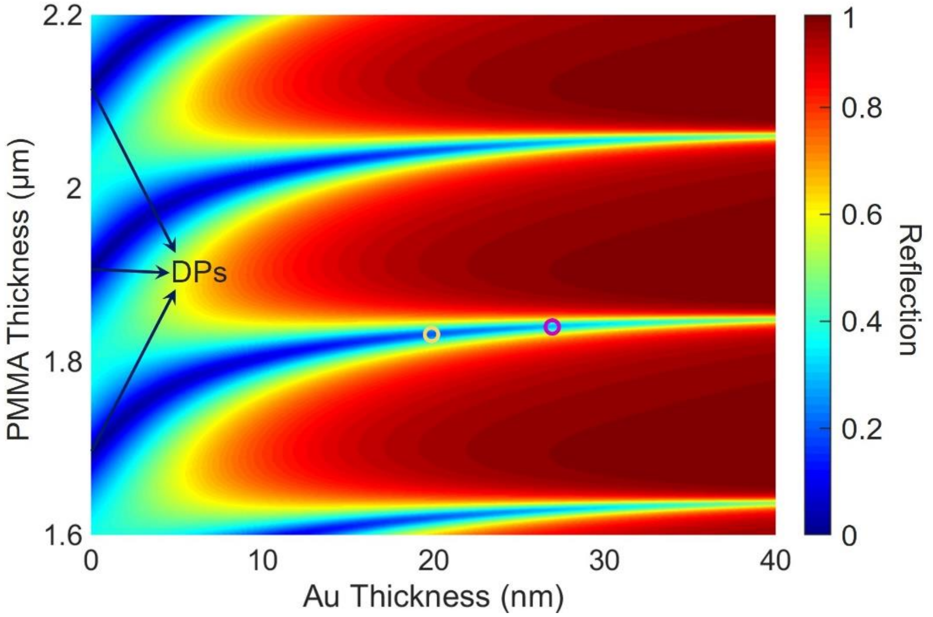

Supplementary Figure 1. Calculated amplitude of reflection at the He-Ne probe wavelength with variable thicknesses of the polymer layer and the Au layers. The diabolic points (DPs) only occur on the axis of 0 Au thickness. The purple and yellow circles mark the reflection dips at the respective Au thicknesses.

While we have proved the superiority of our EP scheme over the DP structure in terms of sensitivity in our manuscript, here we show that in the latter case the increase of reflection as a response of thermal perturbation is still significantly lower than the increase of forward reflection in our EP structure. Supplementary Fig. 2 depicts such a comparison between our EP scheme and a Hermitian multilayer structure with similar parameters and a reflection resonance at the probe wavelength. Although large reflection can be observed away from the resonance in both structures, the increase of forward reflection in our EP structure is almost 5 times the increase of the forward or backward reflection in the Hermitian structure as a response to the same thermal perturbation. One may still raise the concern that the slope of reflection in the Hermitian multilayer structure is limited because the reflection does not reach 0. However, we stress a Hermitian structure with both Au-enhanced reflectivity and yet a DP resonance does not exist in the multilayer normal incidence scheme, and the ultimate slope of forward reflection can only be optimized in an asymmetric non-Hermitian structure, i.e., the EP structure. To further proof the superiority of our EP structure over the Hermitian multilayer structures, we provide in Supplementary Fig. 3 another exemplary comparison with a symmetric structure. The stark contrast of the reflection enhancement under the same thermal perturbation can be clearly observed as well.

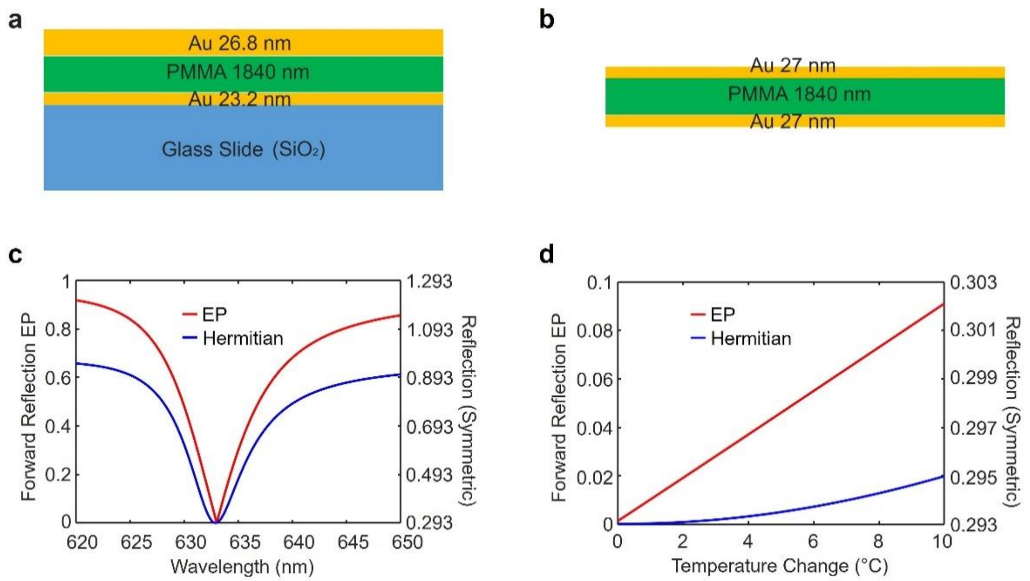

Supplementary Figure 2. Comparison of the sensitivity between the exceptional point structure and a Hermitian multilayer structure. a Schematic of our exceptional point (EP) structure. **b** Schematic of the Hermitian multilayer structure with symmetric Au layers. **c** Spectra of forward reflection of our EP structure and reflection of the Hermitian multilayer structure with resonance at the probe wavelength. **d** Thermal responses of our EP structure and reflection of the Hermitian multilayer structure.

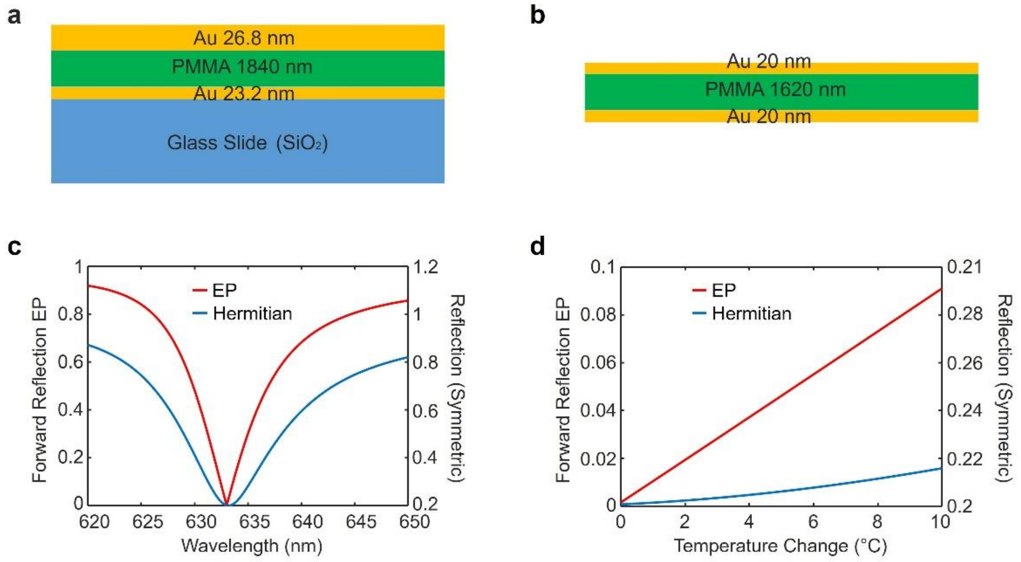

Supplementary Figure 3. Comparison of the sensitivity between the exceptional point structure and a Hermitian multilayer structure with lower reflection at resonance. a Schematic of our exceptional point (EP) structure. **b** Schematic of the Hermitian multilayer structure. **c** Spectra of forward reflection of our EP structure and reflection of the Hermitian multilayer structure with lower resonance at the probe wavelength. **d** Thermal responses of our EP structure and reflection of the Hermitian multilayer structure.

To conclude, we emphasize that the enhanced response of reflection under thermal perturbation arises from the topology of the non-Hermitian EP. Neither a DP structure nor a Hermitian multilayer structure with Au-enhanced reflection can possess the comparable response.

Supplementary Note 3: Direct measurement of the scattering eigenvalue splitting and its equivalence with the combined measurement of the forward and backward reflections. To characterize the behavior of the scattering eigenvalue splitting, a direct measurement of the generalized reflection $|\sqrt{r_\mathrm{f} r_\mathrm{b}}\,|$ is realized by the setup shown in Supplementary Fig. 4. As shown in Supplementary Fig. 4a, a broadband light source is normally incident upon the front surface of the sample, where the forward-reflected power is directed to the backside of the sample (the same spot) by two 50:50 beam splitters and two mirrors. The beam finally reflected from the backside thus contains $|\sqrt{r_\mathrm{f} r_\mathrm{b}}\,|$, which is spectrally characterized using a monochromator. The second mirror and second beam splitter at the backside of the sample are both deliberately tilted at a small angle (~1°) to avoid forming a reflection loop. Since the collected beam by the monochromator contains both the generalized reflection and direct transmission, a separate measurement for transmission is carried out using the same setup where the reflected beam is simply blocked, as shown in Supplementary Fig. 4b. In this transmission measurement, the sample and the transmission path are not altered compared with Supplementary Fig. 4a, so all the settings remain the same (such as the same spot). The generalized reflection is then obtained by subtracting the transmission term, followed by renormalization against the incident power. By replacing the broadband incidence with the He-Ne laser, the thermal response of the generalized reflection at the probe wavelength can also be characterized.

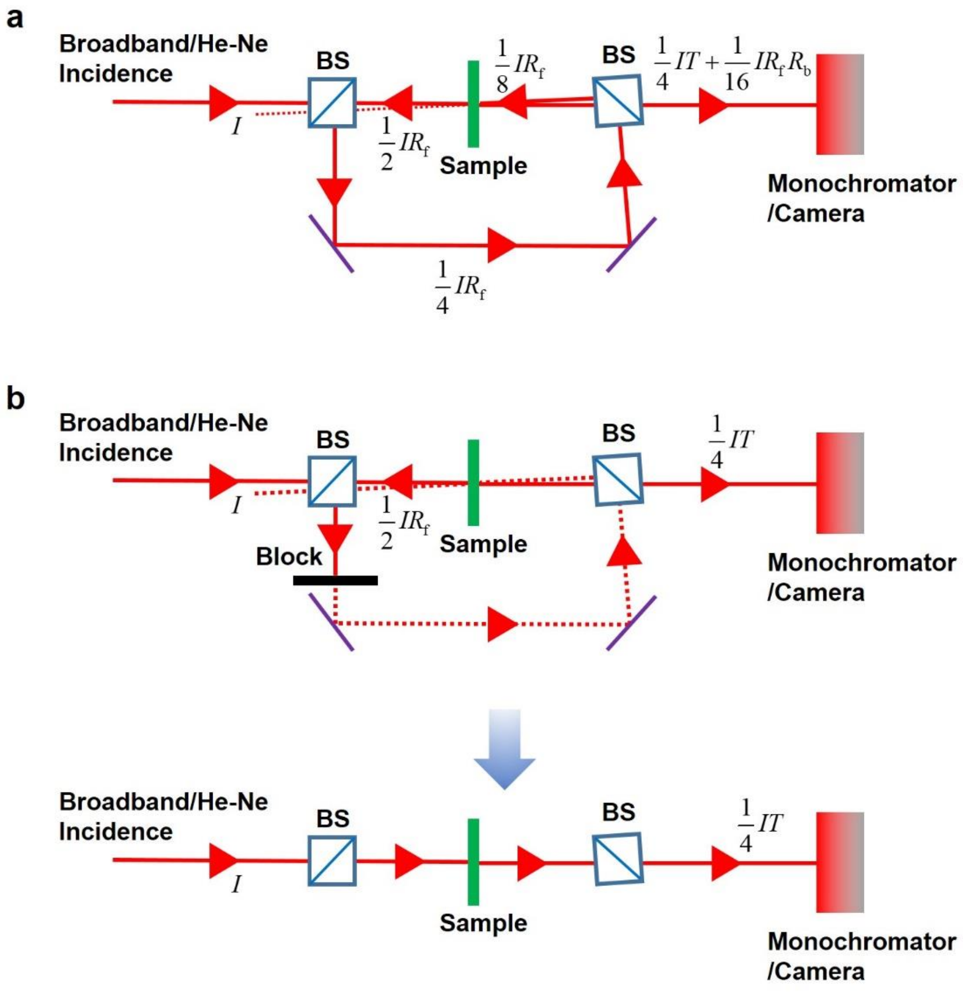

Supplementary Figure 4. Experimental setup for direct measurement of the generalized reflection. a The setup to measure the spectrum and the thermal response of the generalized reflection and transmission. The beam splitter on backside of the sample is deliberately tilted at a small angle (~ 1°) to avoid forming a reflection loop. **b** The setup to measure only the transmission. By blocking the forward-reflected beam, the signal of the general reflection is removed, and thus the setup characterizes only the transmission. The power of each beam is labelled as: I, incident power; T, transmittance; R_f, forward reflectance; R_b, backward reflectance. BS stands for 50:50 beam splitter.

We note, however, this characterization approach is difficult to implement in an intact microscope system, while it can provide the direct and unambiguous measurement of the eigenvalue splitting $\Delta\upsilon = 2|\sqrt{r_f r_b}|$. In contrast, the measurements of the forward and backward reflections ($|r_f|$ and $|r_b|$) can be carried out on a microscope platform at ease. Therefore, if the generalized reflection has the identical behavior as the square root of the

forward reflection multiplying the backward reflection , i.e. $\sqrt{|r_f| \cdot |r_b|}$, the characterization would be conveniently performed in a microscope system. To confirm this equivalence, we measured the spectra of the forward and backward reflections respectively by the Fourier-transform infrared spectroscopy (FTIR), as well as their thermal responses, from which we take the square root of their product to construct the spectrum and the thermal response of $\sqrt{|r_f| \cdot |r_b|}$. In Supplementary Fig. 5, we show the comparison the results with the directly measured generalized reflection (i.e. half of the eigenvalue splitting $|\sqrt{r_f r_b}|$) and its response to the temperature variation using the setup in Supplementary Fig. 4. The slight discrepancy may come from the limited resolution of our monochromator and deviation in alignments compared to FTIR used to separately measure forward and backward reflections.

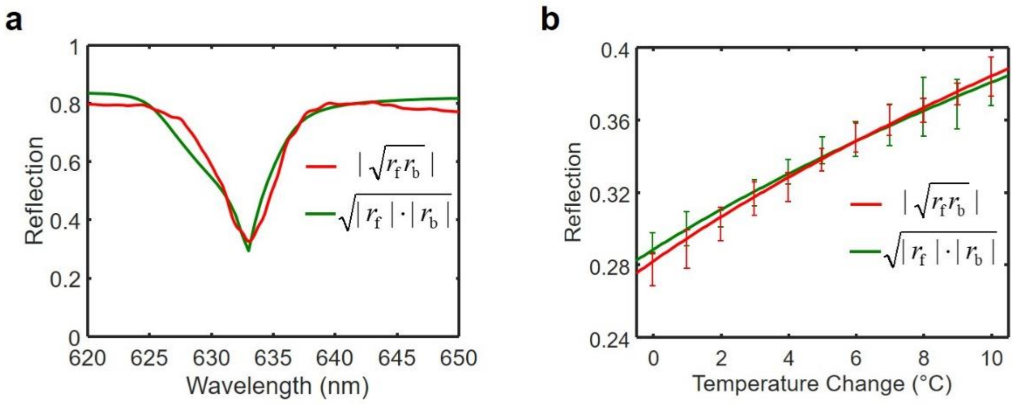

Supplementary Figure 5. Comparison of the directly measured generalized reflection ($|\sqrt{r_f r_b}|$) and the combined measurement ($\sqrt{|r_f| \cdot |r_b|}$). a Comparison in terms of the spectrum. **b** Comparison in terms of the thermal response. The error bars in **b** record the data from 5 separate measurements and indicate the standard deviation. The curves in **b** are square-root fit of the 5 measurements.

Despite the distinct measurement approaches, we observed approximately the same characteristics of the resonant spectrum as well as the thermal response. This is because the multilayer structure in our case is a completely linear system, which means the responses (reflection and transmission) to either forward or backward incidence are independent and satisfy the principle of superposition[2]. Hence, the measurement of $|\sqrt{r_f r_b}|$

in its bilinear form does not contain more information than the combination of two measurements of r_f and r_b. In other words, $|\sqrt{r_\mathrm{f} r_\mathrm{b}}|=|\sqrt{r_\mathrm{f}}|\cdot|\sqrt{r_\mathrm{b}}|$. From another perspective, the transmission and reflection results in theory are obtained in a source-free condition, which means they remain unchanged no matter how the incidence is applied, either single-side incidence (used in the separate measurements) or double-side incidence (the scheme in Supplementary Fig. 4). In our work, therefore, we can apply the separate measurements of the forward and backward reflection to characterize the thermal response for the much simplified experimental setup and its compatibility with a microscope system.

Last but not least, we should note that the square-root derivation of the experimentally observed reflectance or transmittance is always necessary to correctly characterize the scattering matrix (whose entries are reflection and transmission). Such mathematical derivation should not discredit the validity of the experimental data since the enhancement of the sensitivity, as we clarified in the following, does not come from the simple square-root operation. Rather, it arises from the zero forward reflection and large backward reflection (i.e. the highly asymmetric reflection at the probe wavelength), which form the EP together.

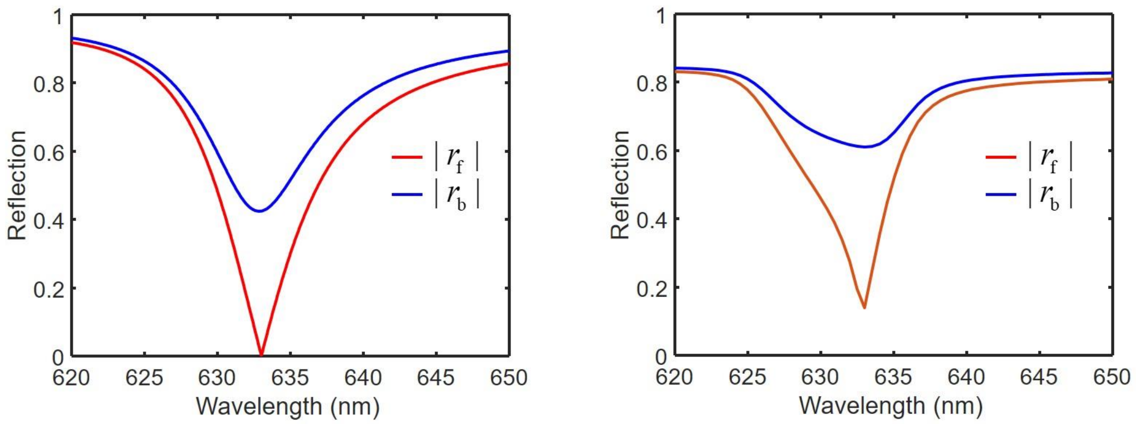

Supplementary Figure 6. Theoretical and measured spectra of the amplitudes of forward and backward reflection coefficients of the exceptional point engineered glass slide. While the forward reflection experiences sharp resonance transition across the exceptional point, the backward reflection varies slowly and maintains at relatively large value.

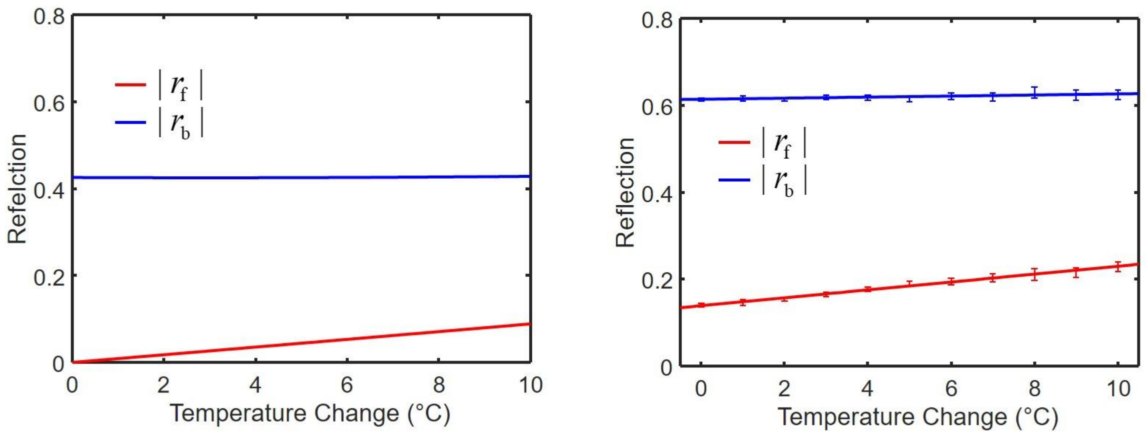

Supplementary Figure 7. The calculated and measured thermal responses of forward and backward reflections. The variation of forward reflection is considerably larger than that of the backward reflection, which acts as the driving force for the sensitivity enhancement. The 10 °C temperature perturbation only renders a change of 0.0032 in the backward reflection even starting from the ideal exceptional point, which is in stark contrast to a large change of 0.0895 in the forward reflection. The error bars record the data from 5 separate measurements and indicate the standard deviation.

Supplementary Note 4: Spatial resolution of thermal mapping. The spatial resolution of thermal mapping is limited by the heat transfer process on the glass slide[3-6], which spreads the heat flux generated from the pulsed laser incidence in the vicinity of the heat source. To solve the temperature distribution and directly visualize the spatial resolution in our multilayer structure, we simulate the temperature distribution where the effect of laser heating is considered as a heat flux resulted from the absorbed laser power density.

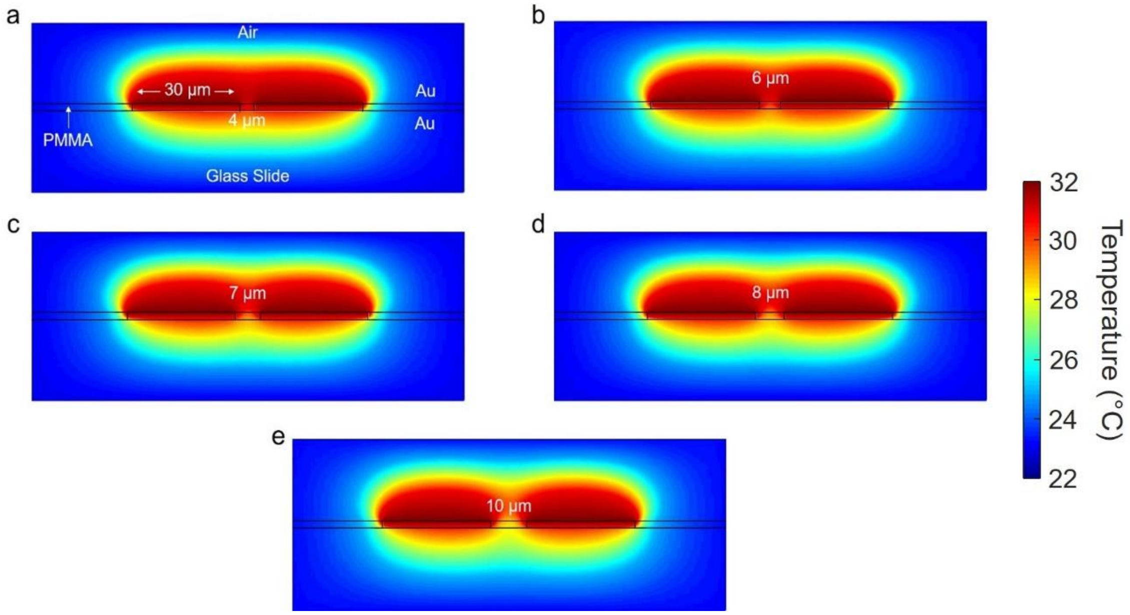

Supplementary Figure 8. Simulation of the temperature distribution under the pulsed laser incidence. Each of the two heat sources is 30 μm wide, mimicking the size of the laser spots. With increasing separation distance from 4 μm to 10 μm (**a**-**e**), the high temperature region splits to two parts from the merging state.

As shown in Supplementary Fig. 8, the temperature exponentially decays within the approximately 4 μm range outside of the heat source. When the separation of two laser spots is as small as 4-7 μm, the high temperature region overlaps leading to spatially a non-distinguishable feature. With increasing of the distance (above 8 μm), the high temperature region starts to separate into two discernible parts. At a further separation of 10 μm, the two heating laser spots can be clearly resolved in thermal mapping. Therefore, we estimate the ultimate spatial resolution of our thermal mapping on the engineered glass slide is at a scale of 10 μm.

Supplementary Table 1. Parameters of material properties for calculations of reflection spectra and thermal response, and simulations of the heat transfer process.

Properties \ Materials	Gold (Au)	PMMA	Glass Slide
Refractive Index *n*	$0.178 + 3.556i$	1.494	1.514
Thermal Expansion α_L ($^\circ C^{-1}$)	1.4×10^{-5}	8.2×10^{-5}	NA
Thermal Conductivity k_T ($W \cdot m^{-1} \cdot ^\circ C^{-1}$)	317	0.19	1.38
Density ρ ($kg \cdot m^{-3}$)	1.93×10^{4}	1190	2203
Specific Heat Capacity c_p ($J \cdot kg^{-1} \cdot ^\circ C^{-1}$)	129	1420	703

Supplementary Note 5: Experimental setup for laser heating. To create a spatially distributed thermal source on the glass slide, we cast the 1064 nm pulsed laser beam on a square-lattice hole array, which is subsequently focused on the glass slide from the back side by a lens with a focal length of 10 cm combined with an objective (MITUTOYO 10X M PLAN APO NIR). Meanwhile, the temperature sensing is realized by the forward reflection of a collimated He-Ne laser incidence. The transmission of the pulsed laser beam is blocked by a filter with a passing band from 500 to 700 nm in order to eliminate its impact on the temperature reading. Supplementary Fig. 9a shows the self-built setup for thermal imaging of laser heating. To obtain the correlation of the temperature variation and the forward reflection of the He-Ne laser beam (Fig. 3b in the main text), the averaged incident power of the pulsed laser beam is varied from 100 $\mathrm{W\cdot cm^{-2}}$ to 600 $\mathrm{W\cdot cm^{-2}}$, and the thermal mapping results are presented in Supplementary Figs. 9b-g.

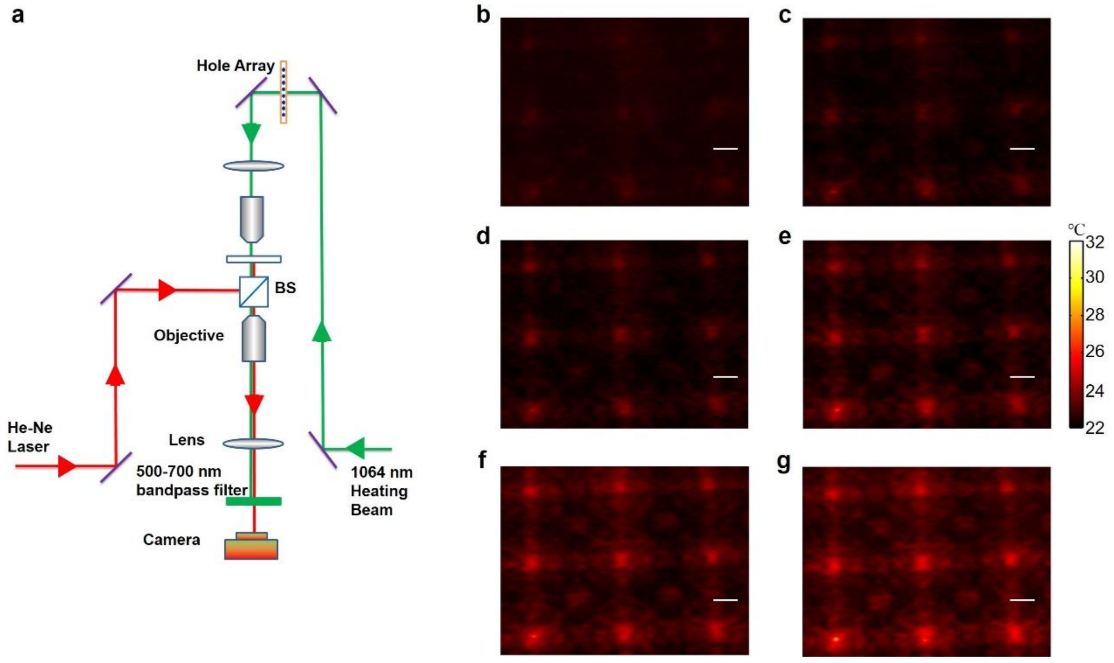

Supplementary Figure 9. Thermal imaging of laser heating. a Experimental setup used to create laser heating using the 1064 nm pulsed laser beam as well as image the corresponding thermal maps through the reflection of the He-Ne laser beam. BS, 50:50 beam splitter. **b-g** Mapped temperature distribution when the power densities of the pulsed laser beam are 100 $\mathrm{W\cdot cm^{-2}}$, 200 $\mathrm{W\cdot cm^{-2}}$, 300 $\mathrm{W\cdot cm^{-2}}$, 400 $\mathrm{W\cdot cm^{-2}}$, 500 $\mathrm{W\cdot cm^{-2}}$ and 600 $\mathrm{W\cdot cm^{-2}}$, respectively. Scale bar in **b-g**, 50 μm.

Supplementary Note 6: Reconfiguration of the optical exceptional point. When the exit medium for the forward incidence is the air ($k_r = k_0$), the optimized EP structure at the He-Ne laser wavelength is realized by 23.2 nm and 26.8 nm-thick Au layers and 1840 nm-thick PMMA layer (Supplementary Fig. 10a). However, the parameters deviate from the EP as the exit medium changes from air to water ($k_r = 1.33k_0$) or PDMS ($k_r = 1.4k_0$) when the glass slide is bonded with the water reservoir (Supplementary Fig. 10b). Therefore, to assure the EP condition at room temperature under a solvent environment, we slightly vary the parameters of two Au layers to 21.4 nm and 25.9 nm while maintain the thickness of the PMMA layer (Supplementary Fig. 10c). Due to the almost equivalent refractive indices between water and PDMS, the region bonded with PDMS also reaches the optimized EP condition after the reconfiguration (Supplementary Fig. 10d).

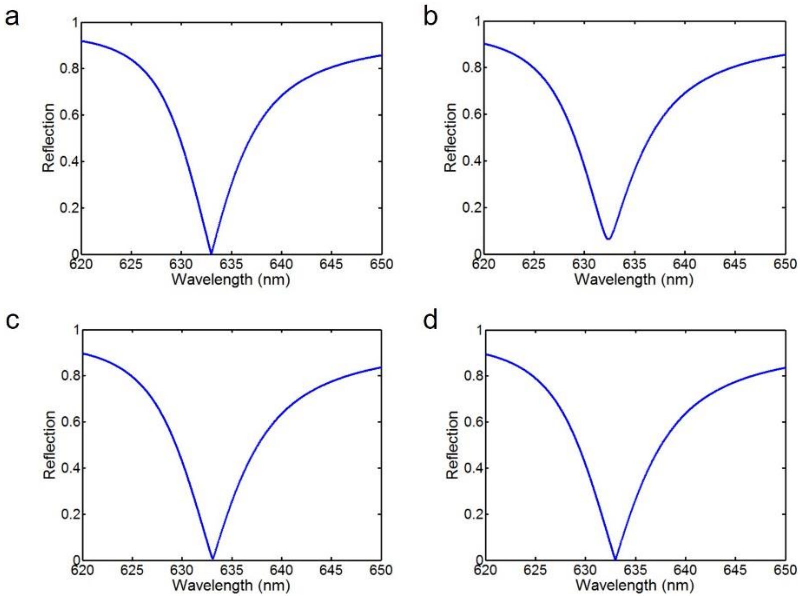

Supplementary Figure 10. Design modification of the exceptional point due to exit medium change. a Spectrum of the optimized optical exceptional point (EP) when the exit medium is air. **b** Deviation from EP when the exit medium changes to water. **c** The recovery of the EP condition after the reconfiguration when exit medium changes to water. **d** The EP condition almost preserves after the reconfiguration when the exit medium changes to PDMS.

Supplementary Note 7: Recording of the thermal conduction process. To detail the process of thermal conduction on the glass slide after the hot water injection, we constantly monitored the forward reflection of the He-Ne laser incidence, which indicates the instantaneous temperature distribution. As presented in Supplementary Fig. 11, the thermal maps are successively captured with a time interval of 10 seconds time interval. This detailed real-time recording further confirms the gradual heat dissipation from the hot water into the ambient PDMS.

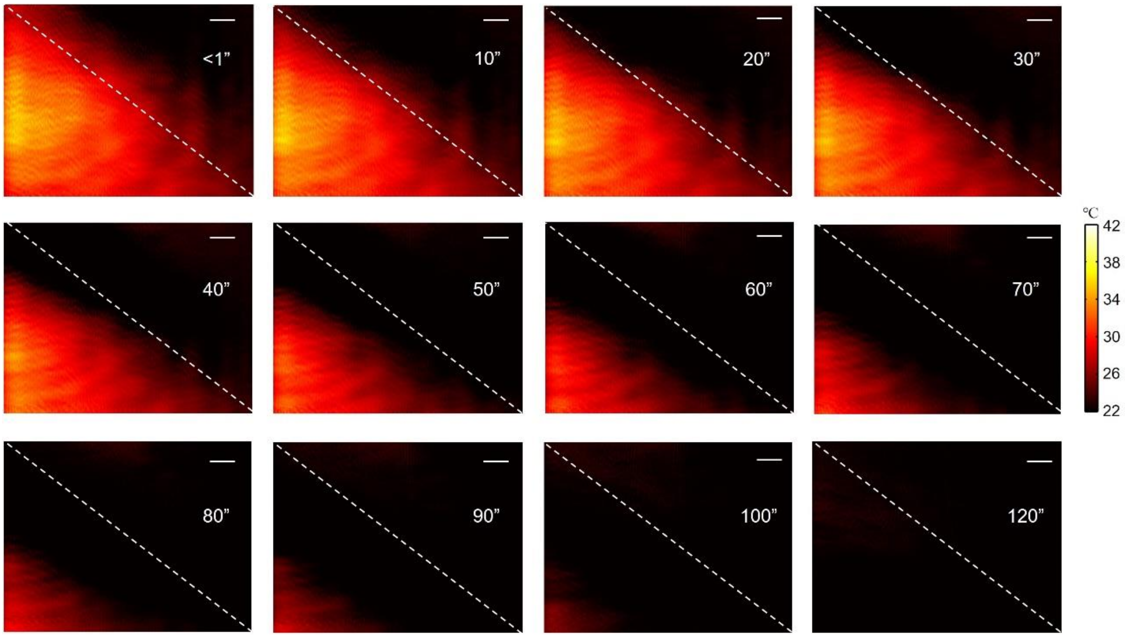

Supplementary Figure 11. Detailed temporal sequence of thermal imaging after hot water injection. White numbers indicate the time when the corresponding images are captured after the hot water injection at an interval of 10 s. Dashed lines represent the boundary between water and PDMS. Scale bars in all panels, 100 μm.

Supplementary References

1. Feng, L., Zhu, X., Yang, S., Zhu, H., Zhang, P.,Yin, X., Wang, Y. & Zhang, X. Demonstration of a large-scale optical exceptional point structure. *Opt. Express* **22**, 1760-1767 (2014).
2. Saleh, B. E. & Teich, M. C. *Fundamentals of photonics*. New York: Wiley 1132-1133 (2007).
3. Burgholzer, P. & Hendorfer, G. Limits of spatial resolution for thermography and other non-destructive imaging methods based on diffusion waves. *Int. J. Thermophys.* **34**,1617-1632 (2013).
4. Burgholzer P. Thermodynamic limits of spatial resolution in active thermography. *Int. J. Thermophys* **36**, 2328-2341 (2015).
5. Chen, Z., Shan, X., Guan, Y., Wang, S., Zhu, J. J. & Tao, N. Imaging local heating and thermal diffusion of nanomaterials with plasmonic thermal microscopy. *ACS Nano.* **9**, 11574-11581 (2015).
6. Tsukamoto, T., Esashi, M & Tanaka, S. High spatial, temporal and temperature resolution thermal imaging method using $Eu(TTA)_3$ temperature sensitive paint. *J. Micromech. Microeng.* **23**, 114015 (2013).
